# Response of an *aspartic protease* gene *OsAP77* to fungal, bacterial and viral infections in rice

**DOI:** 10.1186/s12284-014-0009-2

**Published:** 2014-08-27

**Authors:** Md Mahfuz Alam, Hidemitsu Nakamura, Hiroaki Ichikawa, Akio Miyao, Hirohiko Hirochika, Kappei Kobayashi, Naoto Yamaoka, Masamichi Nishiguchi

**Affiliations:** Faculty of Agriculture, Ehime University, Matsuyama, 790-8566 Ehime, Japan; National Institute of Agrobiological Sciences, 2-1-2 Kan-nondai, Tsukuba, 305-8602 Ibaraki, Japan; Present address; Department of Applied Biological Chemistry, Graduate School of Agricultural Life Sciences, The University of Tokyo, 1-1-1 Yayoi, Bunkyo-ku, 113-8657 Tokyo Japan

**Keywords:** Aspartic protease, Rice, Magnaprthe oryzae, Xanthomonas oryzae pv. oryzae, Cucumber mosaic virus, Tos17 mutant, Probenazole, Salicylic acid, Hydrogen peroxide, Abscisic acidv

## Abstract

**Background:**

Aspartic protease (APs) plays important roles in plant growth, development and biotic and abiotic stresses. We previously reported that the expression of a rice *AP* gene (*OsAP77,* Os10g0537800) was induced by probenazole (PBZ), a chemical inducer of disease resistance. In this study we examined some characteristics of this gene in response to fungal, bacterial and viral pathogens.

**Results:**

To elucidate the spatial and temporal expression of *OsAP77*, the chimeric gene was constructed carrying the structural gene encoding β-glucuronidase (GUS) driven by the *OsAP77* promoter. This construct was introduced into rice and the transgenic lines were tested to analyze gene expression by fungal, bacterial and viral infections. Inoculation with *Magnaporthe oryzae* or *Xanthomonas oryzae* pv. *oryzae* revealed the enhanced GUS activities in vascular tissues surrounding the symptom sites by each pathogen. Moreover, GUS activity also increased after inoculation with *Cucumber mosaic virus* (CMV). Transgenic plants immersed in a solution containing salicylic acid (SA), isonicotinic acid (INA), hydrogen peroxide (H_2_O_2_) or abscisic acid (ABA) showed an increased level of GUS activity exclusively in vascular tissues. RT-PCR analysis showed that *OsAP77* was induced not only by infection with these pathogens, but also after treatment with SA, INA, H_2_O_2_ or ABA. A knockout mutant line of *OsAP77* by the insertion of *Tos17* after inoculation with *M. oryzae*, *X. oryzae* pv. *oryzae* or CMV showed an enhanced susceptibility compared to wild type.

**Conclusion:**

These results suggest that the expression of *OsAP77* is induced by pathogen infection and defense related signaling molecules in a vascular tissue specific manner and that this gene has a positive role of defense response against fungal, bacterial and viral infections.

**Electronic supplementary material:**

The online version of this article (doi:10.1186/s12284-014-0009-2) contains supplementary material, which is available to authorized users.

## Background

Probenazole (PBZ) is a well-known chemical inducer of plant disease resistance (Watanabe et al. [1977]) and has been used to protect rice from blast disease for more than three decades in Japan. A previous study was carried out to elucidate the gene expression profiles of PBZ-treated rice plants (Shimono et al. [2003]). *Aspartic protease 77* (*OsAP77*, Os10g0537800, accession number AK061277) is a PBZ-inducible gene. Shimono et al. ([2003]) found that *OsAP77* induced by 10 fold with the microarray analysis after PBZ treatment (S02370) although it was under the detection level by northern blot analysis. OsAP77, Os10g39260.1, is composed of 395 amino acids (aas) and has a conserved domain of aspartic peptidase (49–390 aas).

Aspartic proteases (APs) are widely distributed in all living organisms, constituting one of the four super families of proteolytic enzymes (Rawlings and Barrett [1999]). APs are expressed in different plant organs, such as seed, grain, tuber, leaf, flower, petal, root and pollen, as well as in the digestive fluids of carnivorous plants (Chen et al. [2009]). Plant APs play versatile roles in protein processing and degradation in different plant organs, as well as in plant senescence, stress response, programmed cell death and reproduction (Simoes and Faro [2004]). Xia et al. ([2004]) reported that *CDR1* (At5g33340) in *Arabidopsis* encodes an AP with a function in bacterial disease resistance. More recently the overexpression of the rice ortholog (*OsCDR1/OsAP5*, Os01g08330) has been reported to induce resistance against fungal and bacterial infections in *Arabidopsis* and rice (Prasad et al. [2009]). Chen et al. ([2009]) identified the total number of 96 *AP* genes in rice and showed the expression data for most of them. However, those of both *OsCDR1*/*OsAP5* and *OsAP77* were not shown in their list because it includes only *OsAPs* of which the expression was detectable in their test. Both APs have signal peptide and a protease motif but are different in active sites: presence/absence in OsCDR1/OsAP77, respectively (Chen et al. [2009]).

Plants are exposed regularly to various environmental stresses including biotic stress caused by a wide range of plant pathogens, such as fungi, bacteria and viruses. However, to our knowledge, there is no report of *OsAP77* in response to plant pathogens. Because of the expression of *OsAP77* was induced by PBZ (Shimono et al. [2003]), it would be reasonable to expect that the *OsAP77* gene is involved in plant defense against pathogens. As a first step to elucidate the role of *OsAP77* gene in disease defense its promoter was analyzed using β*-glucuronidase* (*GUS*) reporter gene system. Transgenic rice plants expressing GUS under the control of *OsAP77* promoter were generated and used to analyze the spatial patterns of *GUS* expression in those plants post fungal, bacterial or viral infection and by treatments with some defense related signaling molecules. Treatments include: 1) infection with blast fungus (*Magnaporthe oryzae*), leaf blight bacterium (*Xanthomonas oryzae* pv. *oryzae*) or *Cucumber mosaic virus* (CMV) and 2) treatment with salicylic acid (SA), isonicotinic acid (INA), hydrogen peroxide (H_2_O_2_) and abscisic acid (ABA). These molecules have previously been used for analysis of abiotic stress (Liu et al. [2009]).

An endogenous retrotransposon *Tos17* has been revealed to be an efficient tool for the functional analysis of rice genes (Hirochika [2001]). The rice mutant lines with the insertion of *Tos17* have become a potent tool for practical use in the systematic analysis of gene function (Kumar and Hirochika [2001]). We used *OsAP77* mutant line disrupted by the insertion of *Tos17* for examining the effect of knockout of *OsAP77*. In this paper we present the GUS activity in vascular tissues of the leaves and the induced expression of *OsAP77* by *M. oryzae*, *X. oryzae* pv. *oryzae*, and several signaling molecules and an enhanced susceptibility of *OsAP77* knockout mutant line to the pathogens.

## Results

### Characterization of putative *cis*-acting elements in the 5′-flanking region of *OsAP77*

In order to better understand the organization of the regulatory region of the *OsAP77* gene, 1,999 bp fragment of the 5′-flanking region of *OsAP77* was isolated by the combinations of primers; OsAP77 pro-5′ and OsAP77 pro-3′, respectively (Table 1). In general, stress-responsive *cis*-acting elements are found in the promoter regions of stress-inducible genes (Hwang et al. [2010]). A promoter motif search was carried out to define putative *cis*-elements in the above mentioned (1,999 bp) 5′ flanking region of *OsAP77*. The potential regulatory elements are the stress-related transcription factor-binding sites including ABA-response elements (ABREs), CCAAT boxes, stress response elements (STREs), the putative *cis*-acting elements responsible for vascular tissue expression (VTRE) and W-boxes (Additional file 1 and Additional file 2). The TATA box was observed at 14 positions upstream of the putative transcription start site in the *OsAP77* 5′- flanking region (Additional file 1 and Additional file 2) (Yang et al. [2011]; Hwang et al. [2010]). While the CAAT box was found at 7 positions (Additional file 1 and Additional file 2), these boxes would serve as basal promoter elements for transcription (Yang et al. [2011]; Hwang et al. [2010]). STRE elements are important for transcriptional activation in response to a variety of stress conditions (Siderius and Mager [1997]). Five STREs were identified in the putative *OsAP77* promoter region. The positions and putative functions of other motifs or elements of the promoter are listed in Additional file 2. These results indicate that the putative promoter sequence of *OsAP77* might be responsive to external factors.

**Table 1:** **PCR primers used in this study**

Primer	Nucleotide sequence^a^
OsAP77 pro-5′	5′-ATGC*CCTGCAGG* ATGACGCAATCAGTCAGACC-3′
OsAP77 pro-3′	5′-ATGC*TCTAGA* AGTATCAACCAGCGATGCTA-3′
∆GUS-5′	5′-AGCTCTAGACTATCCCGCCGGGAATGGTGA-3′
∆GUS-3′	5′-ACGAATTCGGTAGCAATTCCCGA-3′
CMV-R3-cDNA-F-5′	5′-CTGCCTCCTCGGACTTATCC-3′
CMV-R3-cDNA-R-3′	5′-CTGAAACTAGCACGTTGTGC-3′
OsAP-5′	5′-CGGAATTCATGGGAAGGCCAGTGGCAAC-3′
OsAP-3′	5′-CTAGATCTCTAGGAGAGTTTAGTGCAGTC-3′
AP77P-5′	5′-CTCACCGTTGTAGGTAACTG-3′
AP77P-3′	5′-AGTGAGCACGAAATAACTG −3′
Tos17-5′	5′-ATTGTTAGGTTGCAAGTTAGTTAAGA −3′
Actin-5′	5′-GAGTATGATGAGTCGGGTCCAG-3′
Actin-3′	5′-ACACCAACAATCCCAAACAGA-3′

^a^Nucleotides in italic in OsAP77 pro-5′ and OsAP77 pro-3′ indicate *Sbf* I and *Xba* I sites, respectively.

### PCR analysis and GUS staining for selection of transgenic lines

**Figure 1:**
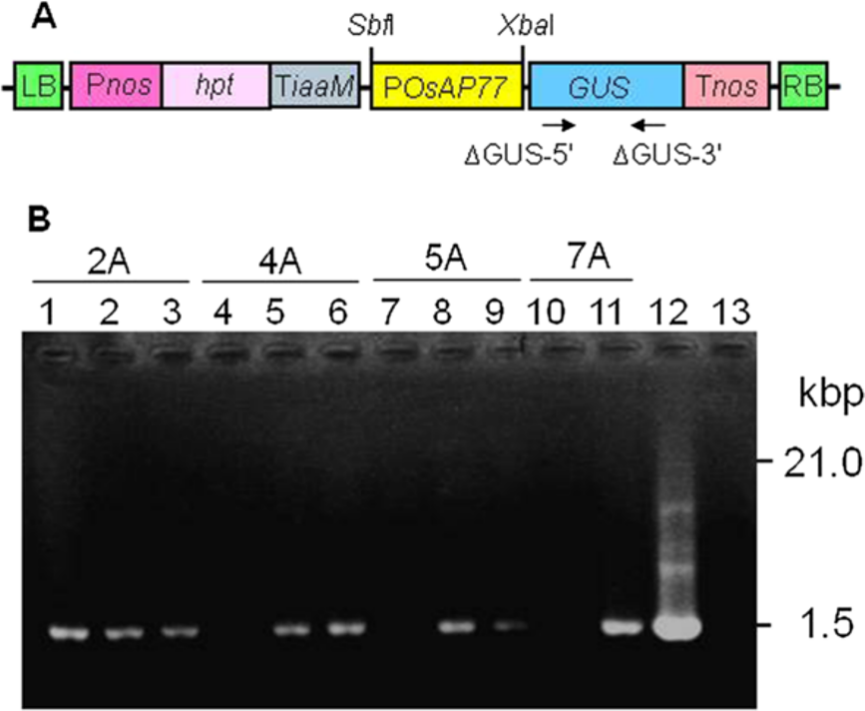
**Plasmid construct and PCR analysis of** ***OsAp77::GUS*** **-transgenic lines (T**_**2**_**) of rice. (A)** Structure of T-DNA region in the expression construct for rice transformation. P*nos* and T*nos*, promoter and terminator of *nopaline syntase* gene, respectively. *hpt*, hygromycin (hyg) phosphotransferase conferring resistance to hyg; T*iaaM*, terminator of *iaa monooxygenase* gene. Arrows indicate the positions of *GUS* gene-specific primers (Table 1). **(B)** Gel electrophoresis of PCR-amplified *GUS* fragment. Lanes 1–3 are derived from line 2A; lanes 4–6, 4A; lanes 7–9, 5A; lanes 10–11, 7A; lane 12, positive control (pBI221 plasmid DNA); lane 13, negative control (non-transgenic line)*.*

Presence of the transgene was examined from 4 T_1_ lines of putative *OsAP77::GUS* transformants. Then, their T_2_ progenies (20 individuals in total) were tested. All primers are listed in Table 1. The PCR-positive individuals were used for further analyses (Figure 1B and Table 2). *OsAP77::GUS* transgene was expressed specifically in vascular tissues of mature leaves of the transgenic lines (2A, 4A, 5A and 7A) at 4-leaf stage (data not shown and Table 2). However, the transgenic rice plants carrying *35S* promoter::*GUS* at the same stage showed GUS activity not only in vascular tissue but also the other tissues (data not shown). Finally, T_3_ plants of the *GUS*-positive line 2A, which shows highest GUS activity compare to the other lines (data not shown), was used for further *GUS* expression experiments in various tissues of mature plants as well as under biotic stress conditions.

**Table 2:** **Analysis of** ***OsAP77::GUS*** **-transgenic rice plants by genomic PCR and GUS staining**

Transgenic line	Plants tested (T2 generation)	PCR-positive plants	Plants stained blue
2A	5	5	3
4A	5	4	2
5A	5	4	2
7A	5	4	3
Control	6	0	0

### Expression of GUS in leaves and roots in 4 week-old plants

To analyze the tissue-specific expression driven by the *OsAP77* promoter, GUS expression was examined in the leaves and roots of 4 week-old transgenic T_3_ plants (Additional file 3). The plants were maintained in the growth chamber. In mature plants at the 1-month-old stage, GUS activity was found in the vascular tissue, but not in mesophyll cells, epidermal cells or guard cells (Additional file 3). These findings show that the promoter is responsible for the precise transcriptional regulation that determines the tissue-specific of the gene in the leaves and roots.

### Induction of *OsAP77* gene expression by probenazole (PBZ)

To confirm whether *OsAP77* is induced in leaves by PBZ, 12-day-old rice plants were treated with PBZ and GUS gene expression was examined. *OsAP77::GUS* expression increased gradually, reached a plateau at 3–5 days and decreased after PBZ treatment. GUS expression was observed only in vascular tissues (Additional file 4).

### Expression of *OsAP77* under biotic stress

**Figure 2:**
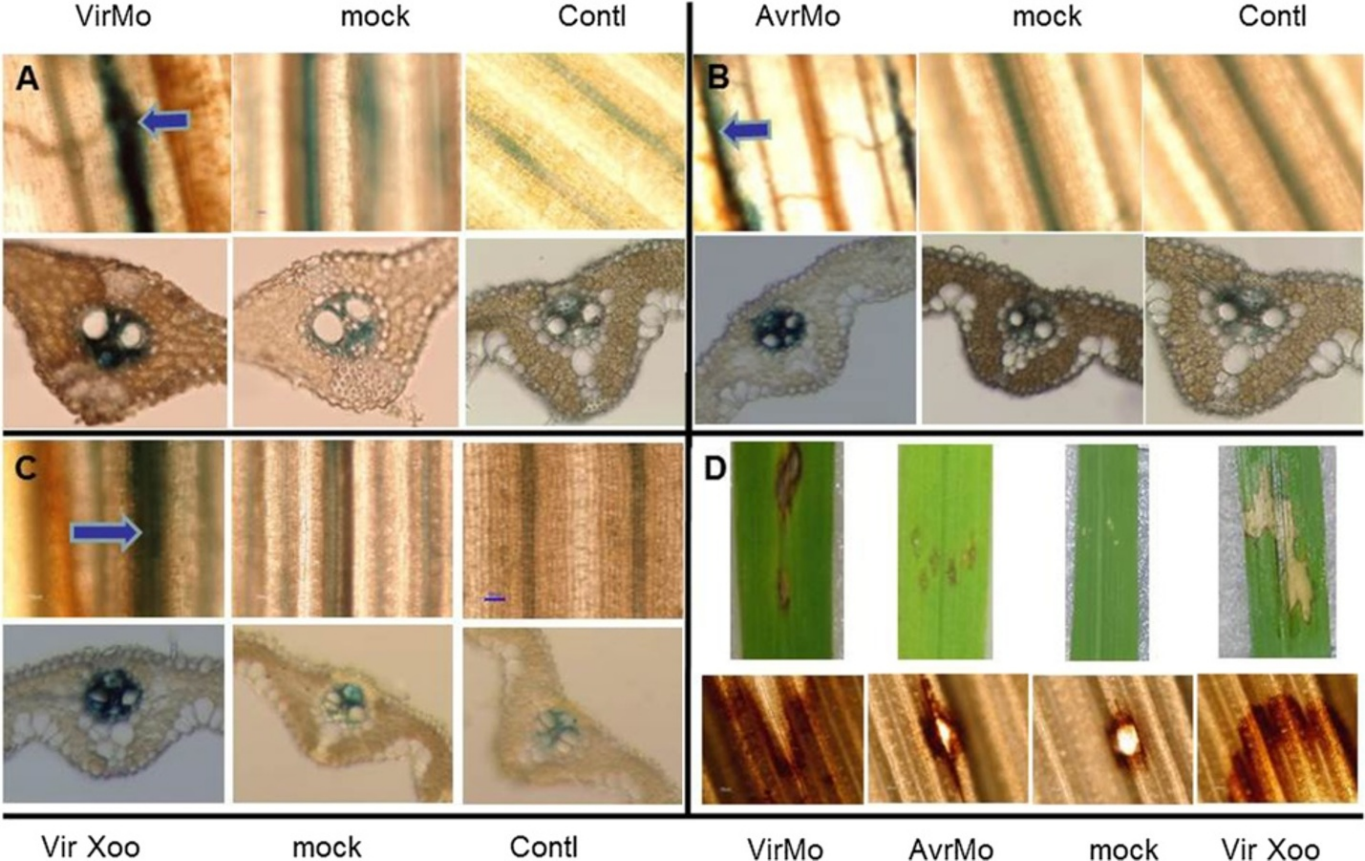
**Expression patterns of** ***OsAP77::GUS*** **after with fungal and bacterial pathogens. (A)** *M. oryzae* (virulent race 001, virMo); **(B)** *M. oryzae* (avirulent race 102.0, avrMo); **(C)** *X. oryzae* pv. *oryzae.* **(D)** Rice blast and bacterial leaf blight disease symptoms (upper panel) and ethanol washed symptoms (lower panel). The detached leaves of T_3_ plant were wounded by a needle-inoculation with/without droplets of conidia suspension (2×10^5^ conidia ml^−1^) and *X. oryzae* pv. *oryzae* at OD_600_ of 0.3. Then 48 h later leaves were subjected to GUS staining. Transverse sections of leaves were shown just below the leaf image. Brown portion is the symptoms by blast fungi and leaf blight bacteria, respectively. The area neighboring the symptoms stained blue, which is clearly shown in the leaf images as well as in transverse sections. virMo with *M. oryzae*; virXoo, with *X. oryzae* pv. *oryzae*; mock, a needle inoculation with water (wounded); contl., no treatment.

To gain more direct evidence whether *OsAP77* is involved in the response to pathogen attack, transgenic and non-transgenic plants inoculated with rice blast fungus, rice leaf blight bacterium or CMV were examined for GUS activity and endogenous *OsAP77* expression, respectively. To precisely define the expression pattern of *OsAP77* after blast fungus infection, we examined the pattern of *OsAP77::GUS* expression in 6-week-old seedlings. *OsAP77::GUS* showed different patterns of *GUS* induction after the infection (virulent and avirulent races) and wounding with symptom (Figure 2A,B and D). Weakly- and strongly-induced GUS activities were observed by mock inoculation (wounding) and a fungal inoculation, respectively (Figure 2B). In the *OsAP77* promoter region, there are several W-boxes which act as a wounding and pathogen responsive element (Additional file 1 and Additional file 2) (Hwang et al. [2010]). Transverse sections were also prepared using the same leaves to examine GUS activity patterns in more detail as shown in Figure 2A and B. However, when inoculated with an avirulent race of blast fungus, *OsAP77::GUS* expression was also induced in the vascular tissues (Figure 2B). This was confirmed by in vitro GUS activity assay (Additional file 5). In the fungus-inoculated leaves, small hypersensitive reaction (HR) lesions were induced at 1 day post infection (dpi) without any remarkable fungal development thereafter, indicating a typical incompatible interaction (Figure 2B and D) (Sasaki et al. [2004]). These data indicate that the expression of *OsAP77* responds to blast fungus infection and wounding.

*OsAP77::GUS* expression levels appeared to increase in response to *X. oryzae* pv. *oryzae* and wounding with symptoms (Figure 2C and D), which was confirmed by in vitro assay of GUS activity (Additional file 5B), suggesting that the expression of *OsAP77* has roles in the defense response to bacterial leaf blight (BLB). Increased GUS activity was detected around the BLB symptom sites as well as the wounding sites (Figure 2C). As shown in Figure 2C, GUS activity was restricted to the vascular tissues around the wounded area, but not in the mesophyll cells, guard cells, epidermal cells and vascular tissues in the BLB and wounded areas. These findings indicate that *OsAP77::GUS* expression is induced specifically in the vascular tissues in the vicinity of the BLB at this stage of growth.

**Figure 3:**
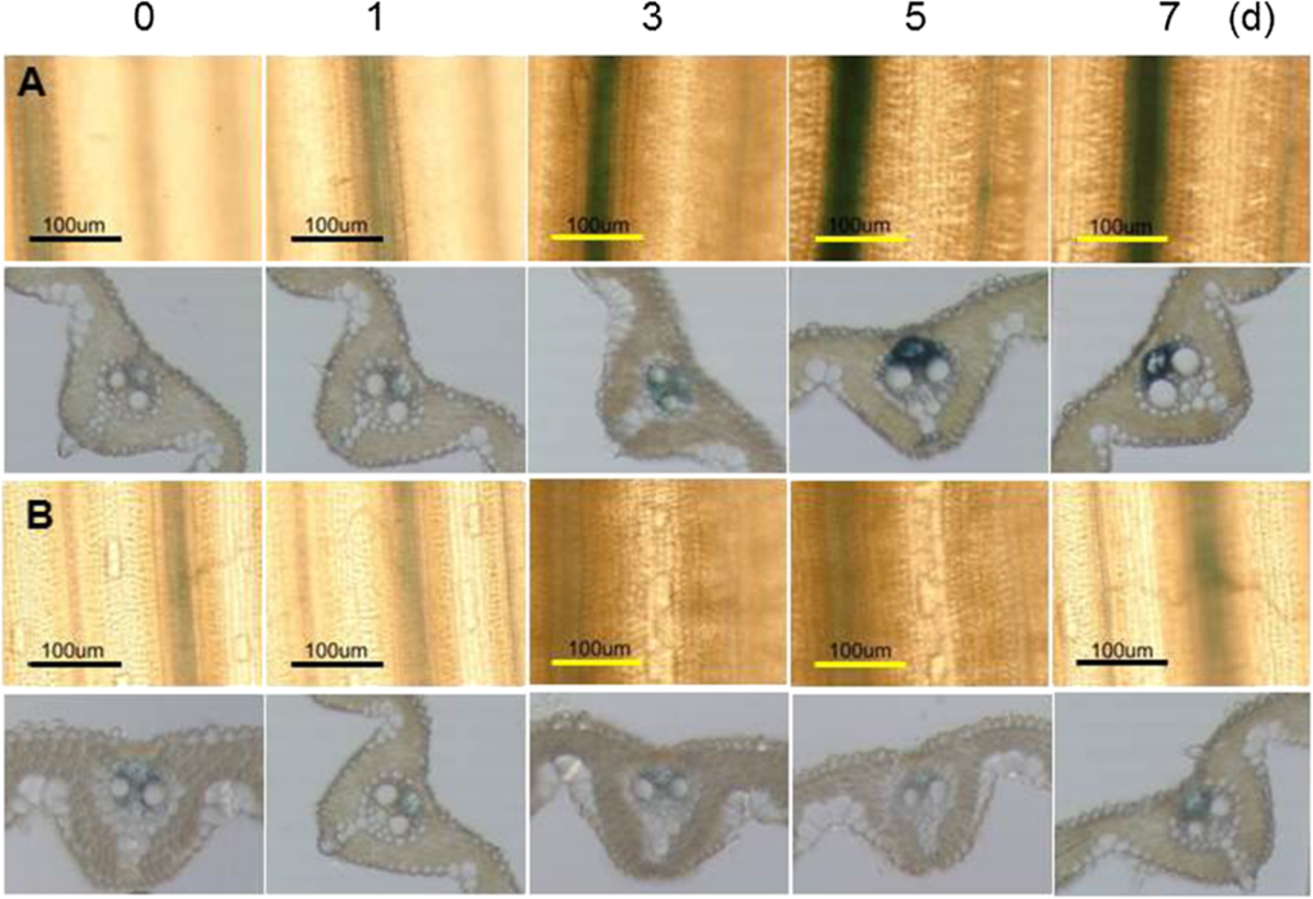
**Expression patterns of** ***OsAP77::GUS*** **after inoculation with** ***Cucumber mosaic virus*** **(CMV). (A)** CMV; **(B)** Mock*.* The 4th young leaves of T_3_ plants were inoculated with CMV. Then leaves were subjected to GUS staining after time course. Transverse sections of leaves were shown just below the leaf image. The GUS activity was analyzed in the leaves treated with indicated duration (d, days) after each treatment.

Increased GUS activity was detected (Figure 3A) 3, 5 and 7 days after CMV inoculation while basal level GUS activity was observed by mock inoculation (Figure 3B). When inoculated with CMV, *OsAP77::GUS* expression was significantly induced only in the vascular tissues and reached its peak after 5 dpi and then declined slowly (Figure 3A). These data suggest that *OsAP77* expression is activated upon CMV infection.

### Expression of *OsAP77* in response to defense related signaling molecules

**Figure 4:**
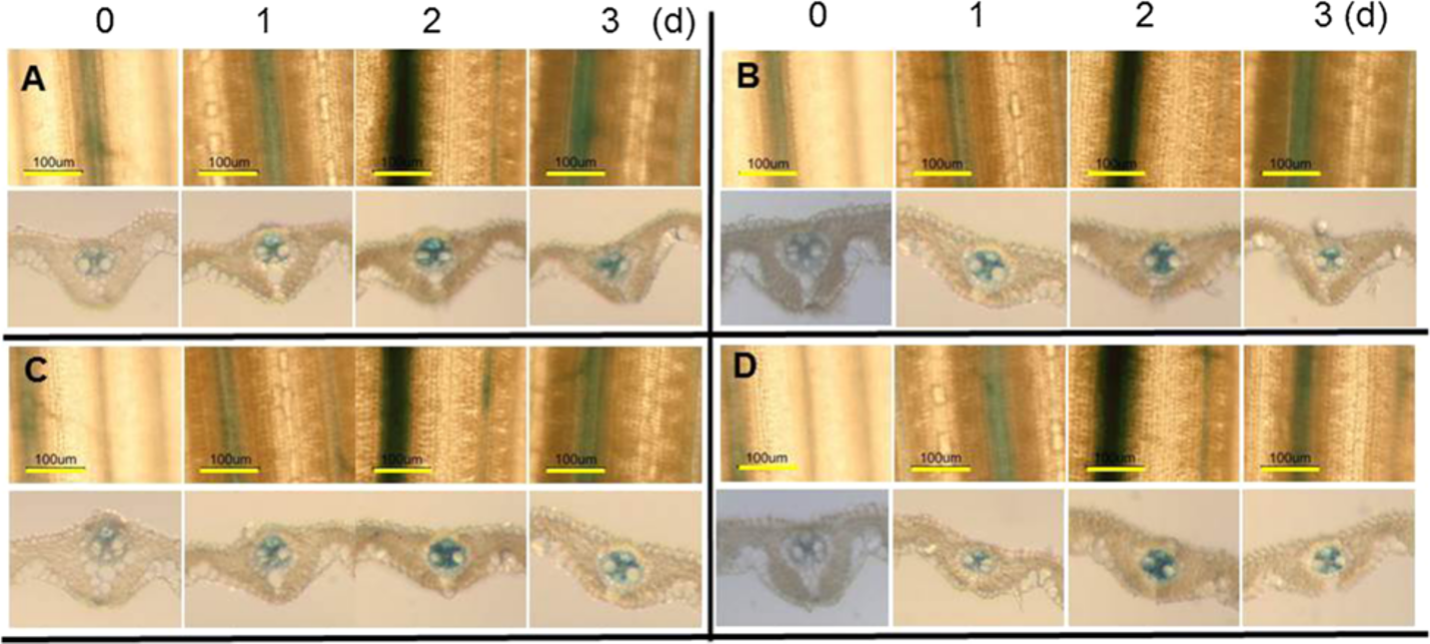
**Effects of signaling molecules on** ***OsAP77::GUS*** **expression. (A)** 10 mM SA; **(B)** 5 mM INA; **(C)** 20 mM H_2_O_2_; **(D)** ABA. The GUS activity was analyzed in the leaves of T_3_ plant treated with indicated duration (days) after each treatment. Rice seedlings at 4-leaf stage, seedlings root were dipped in 3 ml of different solutions and kept at 28°C. The 4th young leaves were cut into 1 cm pieces and used for GUS staining. The GUS activity was analyzed in the leaves treated with indicated duration (d, days) after each treatment.

To determine whether *GUS* expression is induced by defense related signaling molecules, GUS activity in transgenic plants and the expression of endogenous *OsAP77* in non-transgenic plants were analyzed after treatment with SA, INA, H_2_O_2,_ ABA and methyl jasmonate (MeJA). SA could stimulate resistance to plant pathogens, including bacteria, fungi and viruses (Delaney et al. [1994]). The result showed that SA, INA, a biologically active analogue of SA (Chen et al. [1995]), H_2_O_2,_ or ABA induced GUS activity at 1 day post treatment (dpt), reached a peak at 2 dpt and reduced at 3 dpt (Figure 4A,B,C or D, respectively). However, no effects on GUS activity were found after the treatment with MeJA (data not shown).

### RT-PCR analysis of *OsAP77* expression by pathogen infections and defense related signaling molecules

**Figure 5:**
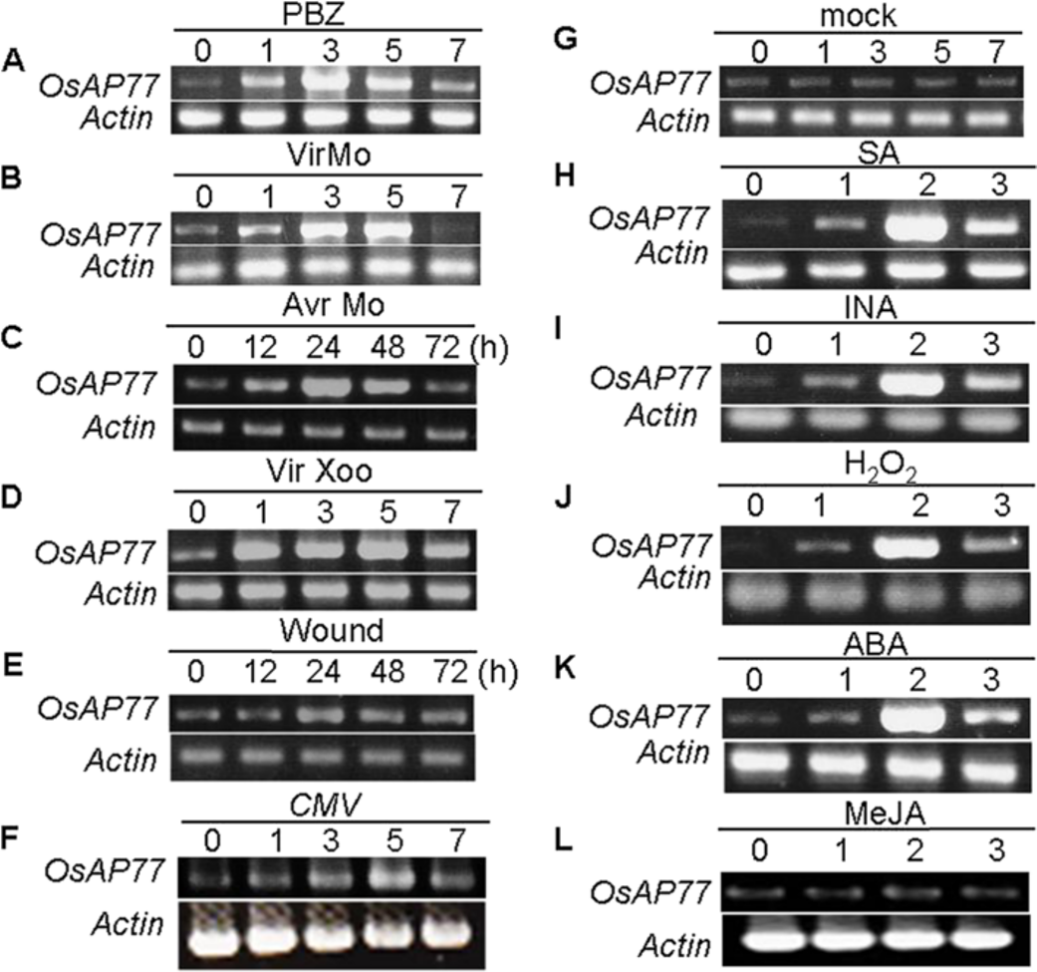
**Expression patterns of** ***OsAP77*** **in response to pathogen infections and treatments with signal molecules.** The treatments of **(A)** probenazole (PBZ); **(B)** *M. oryzae* with virMo; **(C)** *M. oryzae* with avrMo; **(D)** *X. oryzae* pv. *oryzae* with virXoo; **(E)** wound; **(F)** CMV; **(G)** mock; **(H)** SA; **(I)** INA; **(J)** H_2_O_2_; **(K)** ABA and **(L)** MeJA. Numbers represent days after treatment and hours (h) in case of *M. oryzae* with avrMo and wound. Total RNA was extracted from leaves of T_3_ plant at indicted time. The *actin* gene was used as the standard control to show the normalization of the amount of templates in PCR reactions.

To examine *OsAP77* gene expression in relation to some biotic/abiotic stresses, rice plants were inoculated with *M. oryzae*, *X. oryzae* pv. *oryzae* or CMV and to treatment with wounding, PBZ, SA, INA or H_2_O_2_. To understand the induction of endogenous *OsAP77*, wild type rice plants were treated with PBZ, and then used for total RNA extraction followed by RT-PCR. It was found that *OsAP77* reached its maximum expression at 2 dpi (Figure 5A). Moreover, *OsAP77* expression reached a plateau at 3 and 5 dpi with a virulent race of *M. oryzae*, but it was much earlier induced by an avirulent race of blast fungus (Figure 5B and C). An earlier and shorter induction of *OsAP77* expression resulted in successful resistance in case of avrMo. Furthermore, RT-PCR analysis shows gene expression reached a plateau at 1 dpi with *X. oryzae* pv. *oryzae* (Figure 5D). These results indicate that *OsAP77* expression was induced, responding to *M. oryzae* and *X. oryzae* pv. *oryzae* infections. *OsAP77* expression level reached a plateau 5 days after inoculation of rice with CMV (Figure 5F), suggesting that CMV can infect rice plants and induce the expression of *OsAP77. OsAP77* expression was also induced after treatment with SA. The response of *OsAP77* to SA increased gradually, reached a peak at 2 dpt, and then declined (Figure 5H). More or less similar responses in the *OsAP77* gene expression pattern were observed after INA, H_2_O_2_ or ABA (Figure 5I and J, and K). The results shown for *OsAP77* coincide with those using transgenic rice expressing *OsAP77::GUS*.

### Enhanced susceptibility to *M oryzae*, *X oryzae* pv *oryzae* or CMV infection in *OsAP77* knockout mutant plants

**Figure 6:**
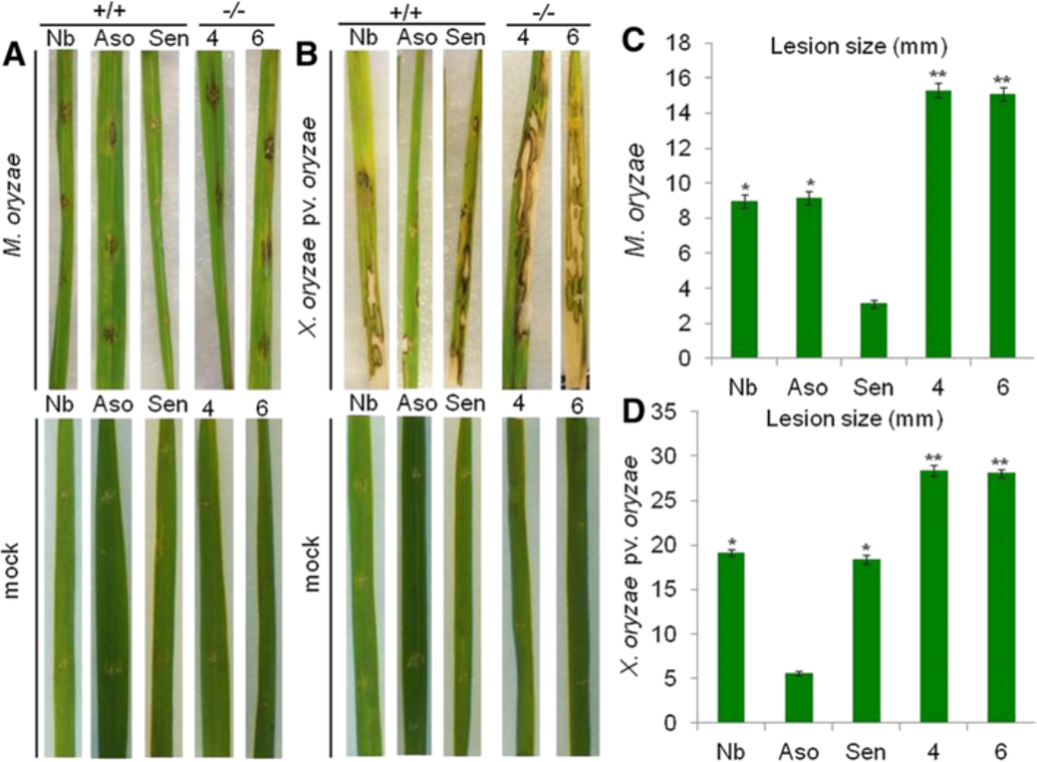
**Symptoms in the knockout mutant line of** ***OsAP77*** **by** ***Magnaporthe oryzae*** **and** ***Xanthomonas oryzae*** **pv.** ***oryzae*** **. (A)** and **(B)** Symptoms developed at 7 days after inoculation (dpi) by *M. oryzae* and *X. oryzae pv. oryzae,* respectively. **(C)** and **(D)** Length of necrotic lesions formed at 7 dpi by *M. oryzae* and *X. oryzae pv. oryzae,* respectively. Nb, Nipponbare; Aso, Asominori; Sen, Sensyou; 4 and 6, *OsAP77*(−/−). M, mock; I, inoculated. Total 5 lesions were measured each in three replicates.

**Figure 7:**
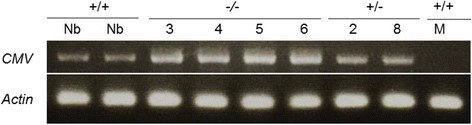
**The accumulation level of** ***Cucumber mosaic virus*** **RNA in the knockout mutant line of** ***OsAP77*** **.** The young leaves were inoculated with *Cucumber mosaic virus*. Total RNA was extracted from the inoculated leaves at 7 dpi. Upper panel showed analysis of CMV after infection. The *actin* gene was used as the standard control to show the normalization of the amount of templates in PCR reactions. Nb, Nipponbare (+/+); 3, 4, 5 and 6, *OsAP77* (−/−); 2 and 8, *OsAP77* (+/−); M, mock in Nipponbare (+/+).

To further investigate the functions of *OsAP77* in defense responses against pathogen infection, M1 progeny of the *OsAP77* mutant line was used for genotyping by PCR with the specific primers (Additional file 6A and B, Table 1). The expression of *OsAP77* was confirmed by RT-PCR. The *OsAP77* expression level was highest in WT, *OsAP77* (+/+) while it was under the detection level in the mutant homozygous plants *OsAP77* (−/−) (Additional file 6C). After the inoculation with either *M oryzae* or *X oryzae* pv *oryzae*, *OsAP77* (−/−) showed more severe symptoms than *OsAP77* (+/+) did (Figure 6A and B). However, the heterozygous plants, *OsAP77* (+/−), showed the intermediate level of symptom severity between *OsAP77* (+/+) and *OsAP77* (−/−) (data not shown). In case of CMV, the accumulation level of CMV RNA was the highest in *OsAP77* (−/−) (Figure 7). These results suggest clearly that *OsAP77* plays positive role to defense against fungal, bacterial and viral infections.

## Discussion

In this paper we analyzed the special expression pattern of *OsAP77* using GUS reporter gene and the function in defense response using its knockout mutant rice. We previously reported that *OsAP77* was PBZ inducible by the microarray analysis (Shimono et al. [2003]). We postulated that this gene might be involved in the defense response against pathogens because PBZ is a chemical inducer of disease resistance (Watanabe et al. [1977]). We took approaches first to analyze *OsAP77* gene expression. Thus we produced the transgenic rice carrying GUS reporter gene under the control of *OsAP77* promoter (Figure 1A) and then examined it under various conditions.

In the mature leaves of 1-month-old plants, *OsAP77::GUS* was expressed in the vascular tissues, but not in other tissues (Additional file 3B), which is why it was under the detection level by northern blot analysis, but was expressed 10-folds higher by microarray analysis after PBZ treatment (Shimono et al. [2003]). In the roots, *OsAP77::GUS* expression was restricted to the vascular tissues of the primary and lateral roots (maturation zone). The plant vascular system includes phloem tissues that transport organic materials and xylem cells that transport water and soil derived nutrients. The detection of cis-elements of promoters distinct for vascular tissues may provide a functional approach to illuminate these mechanisms (Yin et al. [1997]). The GATA motifs, ASL box (GCATC) and box-II (CCCCT) are present in many promoter regions and described as vascular tissue-specific elements (Yin et al. [1997]; Hatton et al. [1995]). These *cis*-acting regulatory elements responding to vascular tissue specific expression (VTREs) were also found in the 5′-flanking region of *OsAP77*. Therefore, *OsAP77::GUS* may be involved in regulating vascular development and function in roots and leaves (Additional file 1 and Additional file 2).

Since *OsAP77* can be induced by PBZ, SA, INA, H_2_O_2_ or ABA, which play important roles in signal defense responses, it is expected that *OsAP77* is also responsive to pathogen infection. The expression of *OsAP77::GUS* was examined under biotic stresses, such as fungal, bacterial or viral infection. It was found that expression of *OsAP77::GUS* as well as *OsAP77* was induced with the fungus, *M. oryzae*, and the bacterium, *X. oryzae* pv. *oryzae* (Figure 2A,B and C). In response to wounding stress, *OsAP77::GUS* exhibited expression patterns parallel to but a little weaker than those induced by *M. oryzae* or *X. oryzae* pv. *oryzae* infection (Figure 2A,B and C). Wounding caused extensive changes in the synthesis of defense proteins, leading to localized resistance at the site of the lesion (McConn et al. [1997]). These observations support the hypothesis that the differential expression of *OsAP77::GUS* indicates the presence of different defense mechanisms in a tissue- and organ-specific manner.

We previously found that CMV-SRO could infect rice without any significant symptoms (Chen et al. [2011]). CMV-SRO is a pseudorecombinant containing CMV-SR RNA1 and 2 and CMV-O RNA3 (Hanada and Tochihara [1987]). Here we present that CMV infection caused the induction of *OsAP77::GUS* expression (Figure 3A). Since in rice there are few virus species which are easy to inoculate manually, this CMV isolate may be very important in studying molecular interaction between rice and viruses as a model system.

Plant hormones such as ABA, JA, ethylene and SA are essential components of different signaling pathways involved in plant defense (Hu et al. [2009]; Vicent and Plasencia [2011]). The expression pattern of *OsAP77* was analyzed after pathogen infections and the treatment by some defense related signaling molecules. We found that *OsAP77* can be induced by SA, INA, H_2_O_2_ or ABA but not by MeJA (Figure 4 and Figure 5). SA plays an important role against pathogens, some insect pests and abiotic stress, while JA does mostly for the defense against insect pests and some pathogens (Vicent and Plasencia [2011]; War et al. [2011]). SA and JA/ET defense pathways interact antagonistically, but emerging records suggest a more complex signaling network inducing both positive and negative interactions (Loake and Grant [2007]). In our study, SA, not MeJA could mediate the up-regulation of *OsAP77::GUS* and endogenous *OsAP77* in rice. Thus this gene may participate in SA pathway in rice. It seems unlikely that the induction of *OsAP77::GUS* expression by pathogen infections is caused by an increased level of SA by pathogen. Although there are some data of this gene in response to six plant hormones including ABA and JA (http://ricexpro.dna.affrc.go.jp/GGEP/), however, it is not clear which plant hormone(s) induced the gene expression. Besides that, there is no data available on vascular tissue specific expression of this gene in rice leaves. There are also no expression data of this gene in the comprehensive list by Chen et al. ([2009]). Our data is the first to clearly show the induced expression of this gene in vascular tissues of leaves by pathogen infections and defense related signaling molecules.

Reactive oxygen species (ROS) play a main role in crosstalk between biotic and abiotic stress responses (Fujita et al. [2006]). Plants produce antioxidants and ROS-scavenging enzymes to control the damage made by harmful molecules (Apel and Hirt [2004]). Pathogen spreading is inhibited by hypersensitive response and cell death requiring coordinated down regulation of ROS-scavenging mechanisms (Apel and Hirt [2004]). H_2_O_2_ is thought to be produced through membrane-bound NADPH oxidases on pathogen infection or wounding, then diffuses into cells and activates various plant defenses (Apel and Hirt [2004]). Furthermore, ROS production is required for ABA-driven stomatal closure, which is closely related to stomatal penetration resistance (Robert-Seilaniantz et al. [2011]). ABA largely regulated abiotic stress, while defense against different biotic stress is specified by antagonism between the SA and JA/ethylene signalling pathways. ABA acts both synergistically and antagonistically with biotic stress signalling, creating a complex network of interacting pathways with cross-talk at different levels (Fujita et al. [2006]; Yasuda et al. [2008]). In our study, H_2_O_2_ was found to induce *OsAP77* expression. However, in our observation SA and ABA both can up-regulated expression of *OsAP77::GUS* and *OsAP77*.

The experiments with the knockout mutant line of *OsAP77* showed that the most severe symptoms by *M. oryzae* and *X. oryzae* pv. *oryzae* was observed in *OsAP77* (−/−) compared to those in *OsAP77* (+/+) (Figure 6). These results showed that *OsAP77* participates in the defense reaction against *M. oryzae* and *X. oryzae* pv. *oryzae.* In the *OsAP77* mutant line, *Tos17* is inserted in the upstream region (−560) of *OsAP77*. This is the case where the insertion of *Tos17* in the upstream of a gene caused the repression of downstream gene expression.

It is not known how OsAP77 functions for pathogen infections in vascular tissues. *Arabidopsis* CDR1 which was up-regulated by SA, has a signal peptide (1–25 aas) from the N-terminal portion, is extracellular AP and accumulates in the intercellular fluid (Xia et al. [2004]). Actually OsAP77 is postulated to have a signal peptide 1–20 aas based on the method of Signal PHMM. The role of CDR1 in defense is suggested to degrade its target proteins/peptides for generation of an extracellular peptide elicitor, which may function as a mobile signal for defense response (Xia et al. [2004]). From this point of view, most plausible scenario for the vascular tissue expression of *OsAP77* may be that OsAP77 accumulates in the extracellular space, sieve-tubes, where host proteins may be present, and functions as protease for processing possible target proteins/peptides into a signal peptide(s). Then this signal molecule may spread through sieve tube to activate host defense response systemically. It remains to determine whether OsAP77 are accumulated in sieve tubes and what are the proteins/peptides targeted for degradation by OsAP77.

Chen et al. ([2009]) reported 96 rice AP genes in rice genome and their expression data in different tissues and under various conditions. However, it includes no expression data on OsAP77 and others including OsCDR1/OsAP5 because their data was from OsAPs, of which the gene expression was detectable in their test (Chen et al. [2009]). OsAP77 is similar to OsCDR1/OsAP5 in that both have signal peptide at the N terminus and protease dmotif and only difference is the presence/absence of active sites in that motif for OsCDR1/OsAP5 and OsAP77, respectively (Chen et al. [2009]). *OsCDR1/OsAP5* would be also expressed in vascular tissues because it has signal peptide as *OsAP77* does. Bi et al. ([2005]) reported three rice APs, OsAsp1, OsAsp2 and OsAsp3. OsAsp1 were most abundantly present in early embryo. OsAsp2 appeared in leaf, callus and immature seeds while OsAsp3 did in leaf, stem and phloem. However they never mentioned any functional involvement in the response to biotic stress. *OsAP77::GUS* was expressed mainly in pollen, vascular tissue of leaf and root meristem (Additional file 3). Thus it would be possible that *OsAP77* has any other function. Recently *OsAP25* and *OsAP37* have been reported to be involved in programmed cell death for development of embryo (Niu et al. [2013]).

To our knowledge, this is the first report on the expression of *OsAP77* induced by fungal, bacterial and viral infections as well as after the treatment with SA, INA, H_2_O_2_, ABA or wounding and the involvement of this gene in defense reaction. The data from our study implicate the importance of comparative analysis in identifying the expression of *OsAP77::GUS* in each tissue and cell type as well as in response to different stresses in order to understand the complexity underlying multiple signaling systems. From this point of view, it is significant and relevant to characterize the transgenic rice over-expressing the *OsAP77*.

## Conclusions

To conclude, this study has shown the role of *OsAP77* in defense response to fungal, bacterial and viral infections.

## Methods

### Plasmid constructs and rice transformation

To generate *OsAP77::GUS* chimeric gene, which contains the *GUS* reporter gene under the control of the 5′-flanking region of *OsAP77*, the *OsAP77* promoter region was isolated using a pair of gene-specific primers designated AP77 pro-5′ and AP77 pro-3′ (Table 1) that carry the extra sequences for *Sbf* I and *Xba* I recognition sites, respectively. The *OsAP77* promoter fragment was amplified by PCR with a DNA polymerase (KOD -Plus-, Toyobo, Osaka, Japan), and the genomic DNA from (*O. sativa* cv. Nipponbare) as a template. The fragment with the accurate sequence for *OsAP77* promoter was then digested with *Sbf* I and *Xba* I and cloned into to a binary vector pSMAHdN627-M2GUS (Hakata et al. [2010]) treated with the same restriction enzymes followed by dephosphorylation (Figure 1). The resulting construct was introduced into *Agrobacterium tumefaciens* strain EHA101 (Hood et al. [1986]) and used to transform rice (*O. sativa* cv. Nipponbare) as described in Toki et al. ([2006]). Putative transformants were selected on a series of selection media supplemented with 30 mg/L hygromycin B (hyg). The integration of the expression cassettes in the plant genome was confirmed by PCR using primers (Table 1) directed against the *OsAP77* promoter (AP77 pro-5′ and AP77 pro-3′) and *GUS* region (∆GUS-5′ and ∆GUS-3′). T_1_, T_2_, and T_3_ progenies were used in the subsequent observation, and analyses. The PlantCARE database (http://bioinformatics.psb.ugent.be/webtools/plantcare/html/) was used for the silico identification of the putative cis-acting elements in the 5′-flanking region.

### Plant materials

Wild type rice (*O. sativa* cv. Nipponbare) and the transgenic lines were used in this study. *OsAP77* mutant line (NC2562) was obtained from National Institute of Agrobiological Sciences (NIAS, https://tos.nias.affrc.go.jp/~miyao/pub/tos17/). The mutant line by the insertion of *Tos17* was selected based on the nucleotide sequence of *OsAP77* as a query as described previously (Chen et al. [2008]) and grown in a growth chamber under the conditions at 25°C, 14/8 hr light and dark. In this mutant line *Tos17* is inserted in the upstream region at −560 of *OsAP77*. Asominori and Sensyo, which are resistant to *X. oryzae* pv. *oryzae* and *M. oryzae* respectively, were gifted by Drs. H. Ochiai and M. Mori at NIAS, respectively. Rice seeds on the MS agar medium with/without hygromycin (30 μg/ml) were incubated in a Petri dish at 27°C under a daily cycle of 16 h continuous light and 8 h dark. One week later the seedlings were transferred to small plastic boxes containing commercial soil for rice cultivation (Iseki, Matsuyama, Japan) and placed in growth chambers where the temperature ranged between 23 and 26°C under 16 h daylight and 8 h dark conditions. Approximately three weeks later the plants were transferred to buckets containing the soil in the growth chambers.

### Histochemical GUS-staining assay

Histochemical GUS staining was performed as previously described by Jefferson ([1987]). For testing the expression level of *GUS* in leaves, fully expanded 4th leaf of each seedling at four-leaf stage was used. Flowers, seeds and roots were directly placed in a 1.5 ml microtube. When fungal or bacterial infection occurred, the infected areas of leaves were cut out after the disease symptoms appeared. The samples were then briefly subjected to vacuum infiltration and kept at 37°C overnight. The samples were treated with fresh 70% ethanol several times, if necessary, until the plant tissues were mostly decolored. Some of the samples were cut into 30-μm thick cross-sections using a microtome (Retoratome REM-710, Yamato Kohki Industrial, Asaka, Saitama, Japan) and observed under a microscope (Labphoto-2, Nikon, Tokyo, Japan). Samples were collected from three transgenic plants (T_3_ progenies of 2A) for each line and only the representative one was shown in Figure.

### DNA extraction and PCR amplification

DNA was extracted from rice leaves by cetyltrimethyl ammonium bromide-based (CTAB-based) extraction procedure (Doyle and Doyle [1987]). Quantity and purity of DNA was measured using a spectrophotometer (GeneSpec I, Hitachi High-Technologies Corporation, Tokyo, Japan). Integration of the expression cassette in the transgenic genome was confirmed by PCR with a pair of primers directed against the *OsAP77* promoter (AP77 pro-5′ and AP77 pro-3′) and *GUS*-coding region (∆GUS-5′ and ∆GUS-3′) (Table 1). Genomic DNA (100 ng) from each transgenic line was used as template. pBI221 vector (Jefferson [1987]) was used as a template DNA for positive control with the *GUS*-specific primers (Table 1). The PCR reaction was for 2 min of 94°C preheating, followed by a 30 cycle amplification program (1 min at 94°C for denaturation, 1 min at 58°C for annealing, and 1 min at 72°C for extension) and a final extension at 72°C for 5 min. The PCR products were analyzed by electrophoresis on a 1.0% agarose gel followed by staining with ethidium bromide.

### Fungal infection

Strains of blast fungus *M. oryzae*, virulent race 001, virMo (MAFF #238988) and avirulent race 102.0, avrMo (MAFF #238991), were obtained from NIAS Genebank, (http://www.gene.affrc.go.jp/index_en.php). The fungal culture and fungal inoculation of rice were carried out essentially as described previously (Shimono et al. [2003]) with slight modifications. A disk of *M. oryzae* was put in the centre of an oatmeal agar medium and incubated in darkness in a growth chamber at 25°C for 15 days. For spore induction, the *M. oryzae* culture was kept under the continuous illumination for 2–5 days. The spores were used at the concentration of 2 × 10^5^ conidia/ml (Shimono et al. [2003]). Detached rice leaves were wounded using needles and a droplet of conidia suspension (2 × 10^5^ conidia/ml) containing 0.05% Tween 20 was applied to the leaf covering the wounded portion (Wang et al. [2007]). The inoculated leaves were incubated under high humid conditions in darkness for 24 h and then transferred to the dark growth chamber at 25°C. Under such conditions, lesions were induced on the leaves. At 0, 1, 3, 5 and 7 days after inoculation, the leaf samples were used for GUS staining as well as for total RNA extraction followed by RT-PCR analysis.

### Bacterial infection

A suspension (500 μl) of *X. oryzae* pv. *oryzae* strain 001 (MAFF #311018, NIAS Gene Bank) was spread on a peptone agar medium and then cultured in darkness in chamber at 28°C for 48 h. The bacteria were collected by adding sterilized ultrapure water and the concentration of bacteria was adjusted to 0.3 OD at 600 nm. For inoculation of rice with *X. oryzae* pv. *oryzae,* rice leaves were wounded with a needle and the wounded regions were immersed in the bacterial suspension. Additionally, a bacterial suspension (20 μl) containing 0.05% Tween 20 was placed the wounded regions of the leaf surfaces. After inoculation, the samples were kept in a growth chamber at 28°C. After 5 days, 50% of the leaf area was covered with bacterial leaf blight symptoms. The samples were then used for GUS staining and RT-PCR.

### Quantitative assay of GUS activity

GUS activity was measured by observing cleavage of the β-glucuronidase substrate 4-methylumbelliferyl β-D-glucuronide (MUG) (Sigma, USA; Jefferson [1987]; Gallagher [1992]). Samples (100 mg) of mock, AvrMo, VirMo and VirXoo infected leaves at different pti were frozen in liquid nitrogen and homogenized for 15 sec. GUS extraction buffer [100 mM potassium phosphate pH 7.8, 1 mM EDTA, 1% Triton X-100, 10% glycerol] (1 ml) was added and mixed by vortex for 10 sec. Centrifuge at 14000 rpm for 10 min at room temperature and the supernatant for each sample (600 μl) was collected and kept on ice for use in MUG assays and for protein quantification. Sample extract (10 μl) was mixed with 400 μl assay buffer (GUS extraction buffer containing 3.2 mM MUG) and incubated in a dark at 37°C. After 60 minutes 590 μl stop buffer (200 mM sodium carbonate) was transferred to the tube. Fluorescence was measured on a RF-1500 Spectrofluorophotometer (Shimadzu Co. Ltd., Kyoto, Japan) at 465 nm when excited at 355 nm. Protein concentrations were determined by the method described by Bradford ([1976]).

### Statistical analysis

Data were subjected to software package used for statistical analysis (SPSS version 16, 2007) and significant differences between individual means established using a Student’s *t* test. Differences at the 5% level were considered significant and denoted by asterisk among different groups.

### Viral infection

Purified CMV-SRO strain (MAFF #104016, NIAS GeneBank) was used as an inoculum because this isolate had been found to infect rice plants (Chen et al. [2011]). The 4th leaves of the 4-week-old seedlings were inoculated with a suspension of virus (10 μg/ml in 10 mM sodium phosphate buffer, pH 7.0) after spreading of Carborundum (600 mesh, Nacalai Tesque Co. Ltd., Kyoto, Japan) on their adaxial surfaces. The leaves were collected from the inoculated plants up to 7 days after inoculation and total RNA extraction followed by RT-PCR analysis using a pair of primers CMV-R3-cDNA-F-5′ and CMV-R3-cDNA-R-3′ (Table 1). PCR program was at 94°C for 2 min, followed by 30 or 35 cycles of amplification (94°C for 45 s, 60°C for 45 s and 72°C for 45 s) followed by the final extension at 72°C for 5 min.

### Treatments by defense related signaling molecules

Twelve-day-old rice seedlings were submerged in water containing 1.0 g/l Oryzemate (24% granules of PBZ) (Meji Seika Pharma, Tokyo, Japan). After 7 days of treatment with Oryzemate, the youngest leaf was used for GUS staining (Shimono et al. [2003]). Rice seedlings at 4-leaf stage were dipped in 3 ml each of solutions containing 10 mM SA, 5 mM INA, 20 mM H_2_O_2_, 10 mM ABA 10 mM MeJA and incubated for 72 h in the growth chamber following the procedure of Mitsuhara et al. ([2008]) with a slight modification. SA was dissolved in sterilized water, while INA was first dissolved in dimethyl sulfoxide (DMSO) and then diluted appropriately with ultrapure water. H_2_O_2_, ABA and MeJA were first dissolved in 99.5% ethanol and then diluted appropriately with ultrapure water, respectively.

### Extraction of total RNA and expression analysis

Total RNA was extracted from flowers, panicles, leaf blades, leaf sheaths and roots of rice plants using TRI reagents kit (Molecular Research Center, Cincinnati, Oh, USA) according to the supplier’s protocol with some modifications. RT-PCR was performed using RNAs isolated from the above-mentioned tissues. Total RNA was treated with RNase-free DNase I (Takara Bio, Ohtsu, Japan) for 30 min at 37°C to remove genomic DNA, and then the cDNA was synthesized from 1 μg of total RNA by using RevertAid reverse transcriptase (Thermo Fisher Scientific, Waltham, MA, USA). A pair of primers, OsAP-5′ and OsAP-3′ (Table 1), was used to amplify a cDNA fragment of *OsAP77*. The amplification was performed at 94°C for 5 min, followed by 30 cycles of amplification (94°C for 1 min, 60°C for 1 min, and 72°C for 72 s). *Actin* transcript was used as internal standard using primers: Actin-5′ and Actin-3′ (Table 1). The RT-PCR experiments were done at least three times.

## Additional files

## Electronic supplementary material

Additional file 1:**Sequence and structural feature of the** ***OsAP77*** **5′-flanking sequence.** The nucleotide sequence of the 5′-flanking region of *OsAP77*. The numbering of nucleotides relative to the putative transcriptional initiation site (+1) is shown on the left of the sequences. The translation start site, ATG, is underlined. The putative TATA box is identified by grey back ground and the putative core promoter (CCAAT) consensus sequences are highlighted in pink background. The W-boxes are highlighted in yellow and the putative *cis*-acting elements responsible for vascular tissue expression are indicated by green. A stress responsive, MeJA-reponsive, ABA-responsive elements, GT-1 and GTGA motifs are in underline, red, light blue, deep blue and dark red, respectively. The locations of cis-elements of interest were identified by using PLACE and Plant CARE databases. The functions and consensus sequences of the corresponding elements are shown in Additional file 2. (AC074196, http://www.ncbi.nlm.nih.gov/). (DOC 44 KB)

Additional file 2:**Putative** ***cis*** **-acting elements and their sequences, positions and possible functions in the 5′-regulatory region of the** ***OsAP77*** **gene.**(DOC 48 KB)

Additional file 3:**Histochemical localization of** ***OsAP77::GUS*** **expression in 28-day-old T**_**3**_**plants.** M, mesophyl cell; Cc, companion cell; Pp, phloem parenchyma cell, X, xylem; Lv, large vascular bundle; Vb, bundle sheath. White arrowhead indicates blue stained area. (JPEG 83 KB)

Additional file 4:**Induction of** ***OsAP77::GUS*** **expression by probenazole.** The activity of *GUS* was analyzed in the leaves of T_3_ plants treated with probenazole. Rice seedlings at 12-days were dipped in 5 ml GUS staining solution at 28°C. Up to 7 days, from the youngest leaves 1 cm cuttings were used for GUS staining. (JPEG 59 KB)

Additional file 5:**Quantitative measurement of GUS activity in leaves.** The detached leaves of T_3_ plants were wounded by a needle-inoculation with/without droplets of conidia suspension (2×10^5^ conidia ml^−1^) of avrMO (A)/virMO (B) or VirXoo (B) at OD_600_ of 0.3. Then at the indicated times leaves were collected frozen by liquid nitrogen. The bars represent measurements averaged across the 3 samples from each transgenic line and repeated three times. Samples with asterisk in each parameter are significantly different: p < 0.05. (JPEG 39 KB)

Additional file 6:**Analysis of progeny of** ***OsAP77*** **mutant line.** (A), (B) Genotyping using two sets of primers AP77P-5′/AP77P-3′ and Tos17-5′/AP77P-3′, respectively. Nb, Nipponbare; 1–9, progeny of the mutant line; H_2_O, negative control. +/+, *OsAP77* (+/+); +/−, *OsAP77* (+/−); −/− *OsAP77* (−/−). (C) The expression level of the *OsAP77* in the mutant M1 progeny and wild type plants by RT-PCR with primers, OsAP-5′/OsAP-3′. Total RNA was extracted from leaves from individual plants and used for RT-PCR. (D) The *actin* gene was used as the standard control to show the normalization of the amount of templates in PCR reactions. (JPEG 33 KB)

Below are the links to the authors’ original submitted files for images.

Authors’ original file for figure 1

Authors’ original file for figure 2

Authors’ original file for figure 3

Authors’ original file for figure 4

Authors’ original file for figure 5

Authors’ original file for figure 6

Authors’ original file for figure 7

## Abbreviations

ABA: Abscisic acid
AP: Aspartic protease
ABRE: Abscisic acid responsive elements
BLB: Bacterial leaf blight
CMV: *Cucumber mosaic virus*
GUS: β-D-glucuronidase
H_2_O_2_: Hydrogen peroxide
INA: 2,6-dichloroisonicotinic acid
MeJA: Methyl Jasmonate
PBZ: Probenazole
SA: Salicylic acid
STRE: Stress responsive-elements
VTRE: Vascular tissue-responsive elements
W-box: Wounding- and pathogen-responsive elements
