# Supplementary material for: Response of an aspartic protease gene OsAP77 to fungal, bacterial and viral infections in rice
Source: Rice (N Y). 2014 Aug 27;7:9. doi: 10.1186/s12284-014-0009-2 (PMC4884039; doi:10.1186/s12284-014-0009-2)
Supplement: Supplementary file 1 — Additional file 1:Sequence and structural feature of the OsAP77 5′-flanking sequence. The nucleotide sequence of the 5′-flanking region of OsAP77. The numbering of nucleotides relative to the putative transcriptional initiation site (+1) is shown on the left of the sequences. The translation start site, ATG, is underlined. The putative TATA box is identified by grey back ground and the putative core promoter (CCAAT) consensus sequences are highlighted in pink background. The W-boxes are highlighted in yellow and the putative cis-acting elements responsible for vascular tissue expression are indicated by green. A stress responsive, MeJA-reponsive, ABA-responsive elements, GT-1 and GTGA motifs are in underline, red, light blue, deep blue and dark red, respectively. The locations of cis-elements of interest were identified by using PLACE and Plant CARE databases. The functions and consensus sequences of the corresponding elements are shown in Additional file 2. (AC074196, http://www.ncbi.nlm.nih.gov/). (DOC 44 KB) [file 12284_2014_9_MOESM1_ESM.doc]

| **Additional file 1** | |
| --- | --- |
| -1999 | ATGACGCAATCAGTCAGACCGACTTATGTAAAGGGAAGGGGGAAAAAGCAAAATAAGAAG |
| -1939 | CTGGAGCTTAAAAAACTACGGCGGCTAAATACAAACTCCAAGCAGCGGGAAGGGGCCGAG |
| -1879 | CCACGTTACACTCCTAACAAGTCCCTAAAAACGGCAACGCCAGCTAAAGACTATAGATGG |
| -1819 | CCCTCCTGAGTGGATGGACTCCAGAACCACTGAGACCGAAGAGAACACAAAGTCGAAAAA |
| -1759 | CCTAGAGTTCAGTTCCTTCCACAGCTGCCAAGAGACCAGGAGCACCAGGAAGTCGAAGCT |
| -1699 | AGCGCGGAGATGCTCAGGTTAGCAGGCCCTGAAGGACGGCCACCAGTCCTAGAACCAGCT |
| -1639 | GCCACGGGGGAGAGAGGGCATCCCAGCTAGGAGGCGACAGCACACAGAGCCAGACCTACT |
| -1579 | GAGCAAACACATAGTCGAGGAGGATGTGGTTCGCCGTCTCAAGATCCTGAGTGCAAAAAG |
| -1519 | GGCAGGCCGAGTGACTATCGATGCCATGCTTCTGGAGGAGATCAGTCGTCCAACAACGCT |
| -1459 | GTTGGAATGCAAACCAAAGGAAGAACCTGCATTTAGCAGGACCCTTTACGTGCTAGAGAA |
| -1399 | GATCAGCTCAGGCAAATGAATGCTGGCCATGGAAGAAAGCCTGGTATGCCGAACGCGCAG |
| -1339 | AGTAGCGCTGGTCATACGTCCAACACCAAATGAGACGATTCTCGACACCCGGTGAGAGCT |
| -1279 | GTACGTGAAGCACCGCATCCCAAATAAGGAGAAACTGGGAGATGACTGGGACAGTGAGGG |
| -1219 | CACCGCGGATGTCAGAGACATAGGAGTTGTTCGCCAGACCAGAAGCAACCGTGCGTGAAC |
| -1159 | CTGGAAGACGCTGGGGAACAACGAAGAGGAGATCTGGAGCCAAGGACGCCACCGAGCGGC |
| -1099 | CATCGATCCATTGGTCGGACCAGAAGAGCATAGACTCACCATTTCCCGGCACGAAGAAAG |
| -1039 | TGGATGCCGCGAACATGTCGGAGACGGTCTGCTTGACTGGGGACTTCAGGCCATCCCAGC |
| -979 | AGGTTTATTTTTTCGCGAACGCGTAAAAAGATTGCACATCAATATATTAGAAGAAAAGAG |
| -859 | AAAATAAGAAGCTGAAGCTTAAAAAACTACGGCGGCTAAATACAAACTCCAAGCAGCGGG |
| -799 | AAGGGGCCGAGCCACGCTACACTCCCAACTAGTCCCAAAAACGGCAACGCCAGCTATAGA |
| -739 | CCATAGATGGCTCTTGACTAGGTTTATTAATTTTTACTAGAGAATTAAAGGTTCAAAATG |
| -679 | TAATGTTATCCTCACCGTTGTAGGTAACTGTATCGATCGGTGACGTTTGTGTTTGAATGG |
| -619 | AACTCCTAGCAGCAACAGGGCCACGTTTGCTCTGCGAACAGGTCGTAAGTACAACGTACG |
| -559 | CACTGAATTTCTTCACCTCTCCAGCCAATCAACGGTGGGCCACATTGCTGAACATATAAA |
| -499 | TTCCACGTTGCTGAATAAGCACATTGCCGTCAATATATTAAACTTGATCCATTGGAGATA |
| -439 | TAGTATATTTATATAGAGAATCAACGACTCCATGAAGGCTCAAGTGTCTCATCCATTTCA |
| -379 | CAGTTATTTCGTGCTCACTTATTAACGATTATGAGTTCTTATCTCATTTTTCCTAATTCA |
| -319 | CATCTTTCGTTTTTGGTACGCACGCTTTTCAAATTATTCAATGGTGTGTTTTTTTTTAAA |
| -259 | AAAATTTCTATGCGAAAGTTGTTTTAAAAAAGTATTAACTATTTTTATACGAAAGTTGTT |
| -199 | TCAATAGTTAATACTTAATTAATCGTGCAATAATGCATGTTCCGTTTTATGTGCCGGGAG |
| -139 | GCAACCCCTCCTCCCGAACACACCCAGAAGTTAACATCAATTTATTCAGTCCTATGTTCT |
| -79 | CGTGGTTCGTCCATGCAAACCAATCAGCTGCAGCTACTGTCCCTATATAAACCTAGCTAC |
| -19 | TCATCAACTTCGATCATAA+1 tcaagcatcaatccctcattagcatcgctggttgatactaaactaacagtagccataatca |
| +61 | Tgggaaggccagtggcaacgctgctggtgctgtgcttcatctctgtcacggcgcgcgccgccgcgttccgcgtgcacggccgcctcctcg |
| +151 | ccgacgccgcgacggagggaggcgccgtcgtgcccatccactggacccaggcaatg |
