# Supplementary material for: Response of an aspartic protease gene OsAP77 to fungal, bacterial and viral infections in rice
Source: Rice (N Y). 2014 Aug 27;7:9. doi: 10.1186/s12284-014-0009-2 (PMC4884039; doi:10.1186/s12284-014-0009-2)
Supplement: Supplementary file 2 — Additional file 2:Putative cis -acting elements and their sequences, positions and possible functions in the 5′-regulatory region of the OsAP77 gene.(DOC 48 KB) [file 12284_2014_9_MOESM2_ESM.doc]

**Additional file 2: Putative *cis*-acting elements and their sequences, positions and possible functions in the 5′-regulatory region of the *OsAP77* gene.**

| ***Cis*-element** | **Positions** | **Function** |
| --- | --- | --- |
| ABRE (Hwang et al. 2010) | ACGT:(+): -1877 to -1874, -1412 to -1409, -1324 to -1321, -1277 to -1274, -884 to -881, -637 to -634, -597 to -594, -566 to -563 -495 to -492 (9) | The abscisic acid responsiveness |
| CAAT-box  (Yang et al. 2011; Hwang et al. 2010) | CAAT: (+): -1993 to -1910, -940 to -937, -903 to -900, - 534 to -531, -459 to -456, -281 to -278, -172 to -169, -102 to -99, -59 to -56, (-): -1090 to -1087, -949 to -946, -516 to -513, -477 to -474, -449 to -446, CCAAT: (+) -535 to -531, -60 to -56, (-) -1090 to -1086, -449 to -445 (19) | Common *cis*-acting element in promoter and enhancer regions |
| GT-1 motif  (Tarzaghi and Casmore 1995) | GAAAAA: (+): -1958 to -1953, -1765 to -1760, -869 to -664, (-): -971 to -966, -333 to -328 (5) | Pathogen, SA and salt responsive elements |
| GTGA motif  (Rogers et al. 2001) | GTGA: (+): –1509 to -1506, -1288 to -1285, -1275 to -1272, -1226 to -1223, -1165 to -1162, -640 to -637, (-): -1064 to -1061, -668 to -665, -547 to -544, -382 to -379, -365 to -362, -322 to -319 (12) | Cis-regulatory elements required for pollen expression |
| STRE (Hwang et al. 2010) | AGGGG: (+): -1964 to -1960, -1889 to 1885, -873 to -869, -797 to -794, (-): -135 to -131(5) | Stress responsive elements |
| TATA-box  (Yang et al. 2011; Hwang et al. 2010) | TATAAAT: (+): -505 to -499, (-): -433 to -427, TATTAAT: (+): -715 to -709,TATATAA: (+): -36 to -30, (-): -431 to -425, TTATTT: (+): -975 to -970, -376 to -371, (-): -1949 to -1944, -1258 to -1253, -858 to -853 (10) | Core promoter element around −30 of transcription start |
| TGACG-motif  (Liu et al. 2009) | TGACG: (+): -1998 to -1994, -639 to -635, (-): -453 to -449 (3) | Methyl Jasmonate responsive elements |
| VTRE (Yin et al. 1997; Hatton et al. 1995) | GATA: (+): -443 to -440, (-): -1504 to -1501, -673 to -670, -649 to -446, -340 to -337, GCATC: -1622 to -1618, -1265 to -1221, CCCCT: (+): -135 to -131, (-): -1964 to -1960, -1889 to 1885, -797 to -794, -873 to -869 (12) | *Cis*-acting regulatory element required for vascular tissue expression |
| W-box (Hwang et al. 2010) | TGAC: (+): -1998 to -1995, -1504 to -1501, -1217 to -1214, -1006 to -1003, -908 to -905, -725 to-722, -639 to -636, (-): -1987 to -1984, -1329 to -1326, -1209 to -1206, -471 to -468 (11) | Wounding and pathogen responsive elements |

Figures in parenthesis represent the total number of sites. (+) current strand and (-) opposite strand

**References**

Hatton D, Sablowski R, Yung MH, Smith C, Schuch W, Bevan M (1995) Two classes of cis sequences contribute to tissue-specific expression of a *PAL*2 promoter in transgenic tobacco. Plant J 7:859-876

Hwang JE, Hong JK, Lim CJ, Chen H, Je J, Yang KA, Kim DY, Choi YJ, Lee SY, Lim CO (2010) Distinct expression patterns of two *Arabidopsis* phytocystatin genes, *AtCYS1* and *AtCYS2*, during development and abiotic stresses. Plant Cell Rep 29:905-915

Liu Y, Gao Q, Wu B, Ai T, Guo X (2009). *NgRDR1*, an RNA-dependent RNA polymerase isolated from *Nicotiana glutinosa*, was involved in biotic and abiotic stresses. Plant Physiol Biochem 47: 359-368

Rogers HJ, Bate N, Combe J, Sulllivun J, Sweetman J, Swan C, Lonsdale DM, Twell D (2001) Functional analysis of cis-regulatory elements within the promoter of the tobacco late pollen gene *g10*. Plant Mol Biol 45: 577-585

Terzaghi WB, Cashmore AR (1995) Light-regulated transcription. Annu Rev Plant Physiol Mol Biol 46: 445-474

Yang H, Wang M, Gao Z, Zhu C, Guo X. (2011) Isolation of a novel *RNA-dependent RNA polymerase 6* from *Nicotiana glutinosa*, *NgRDR6*, and analysis of its response to biotic and abiotic stresses. Mol Biol Rep 38:929-937

Yin Y, Chen L, Beachy R (1997) Promoter elements required for phloem-specific gene expression from the RTBV promoter in rice. Plant J 12(5):1179-88
