# Supplementary figures and images for: Response of an aspartic protease gene OsAP77 to fungal, bacterial and viral infections in rice
Source: Rice (N Y). 2014 Aug 27;7:9. doi: 10.1186/s12284-014-0009-2 (PMC4884039; doi:10.1186/s12284-014-0009-2)

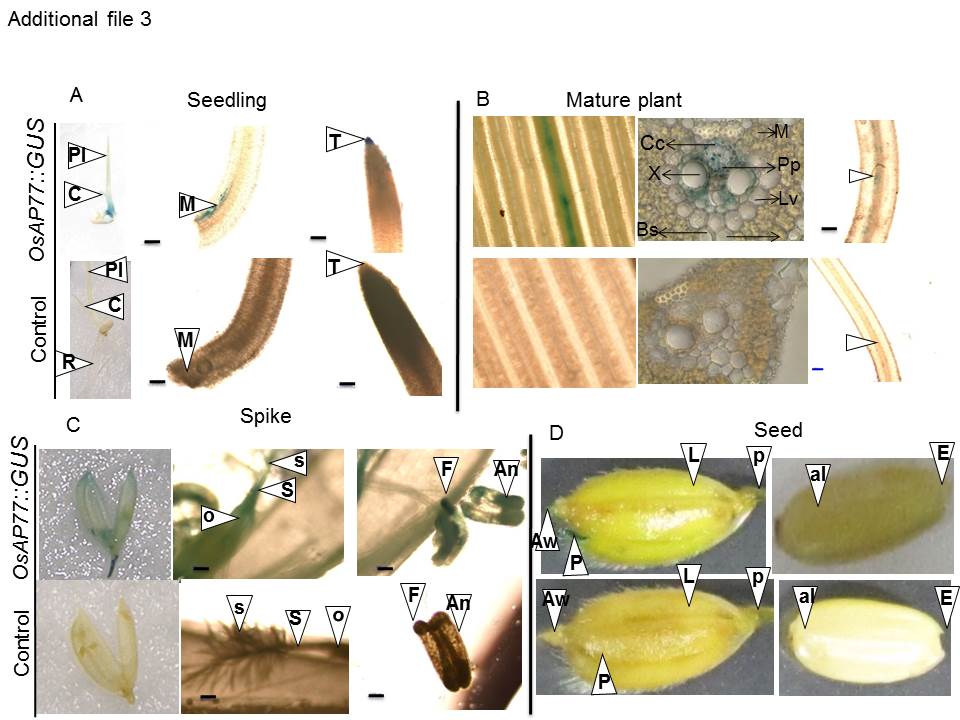

Supplement: Supplementary file 3 — Additional file 3:Histochemical localization of OsAP77::GUS expression in 28-day-old T3plants. M, mesophyl cell; Cc, companion cell; Pp, phloem parenchyma cell, X, xylem; Lv, large vascular bundle; Vb, bundle sheath. White arrowhead indicates blue stained area. (JPEG 83 KB) [file 12284_2014_9_MOESM3_ESM.jpeg]

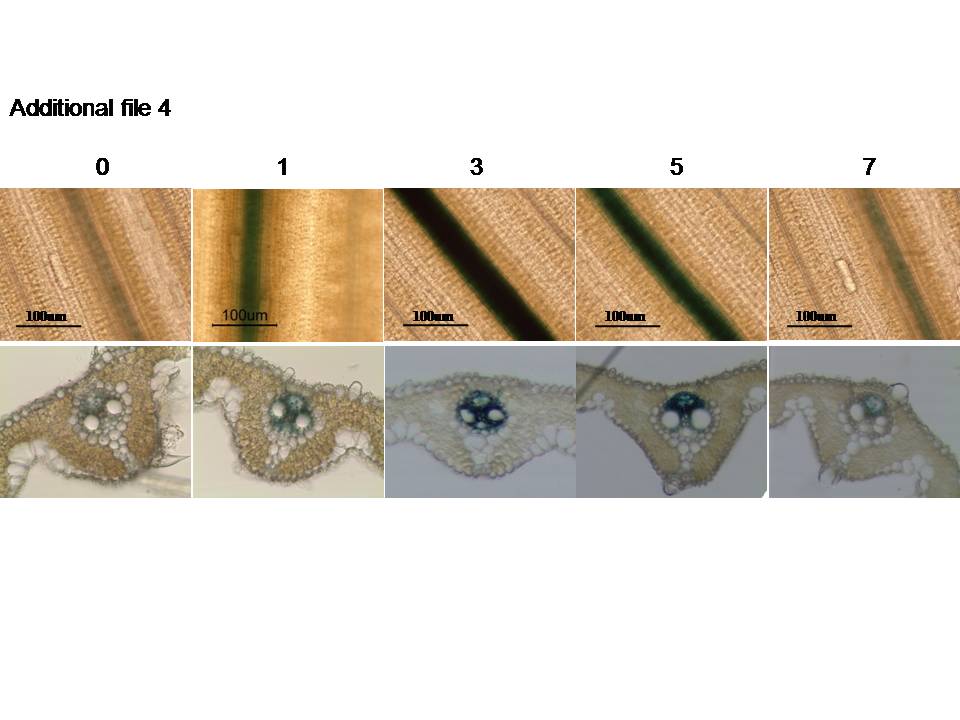

Supplement: Supplementary file 4 — Additional file 4:Induction of OsAP77::GUS expression by probenazole. The activity of GUS was analyzed in the leaves of T3 plants treated with probenazole. Rice seedlings at 12-days were dipped in 5 ml GUS staining solution at 28°C. Up to 7 days, from the youngest leaves 1 cm cuttings were used for GUS staining. (JPEG 59 KB) [file 12284_2014_9_MOESM4_ESM.jpeg]

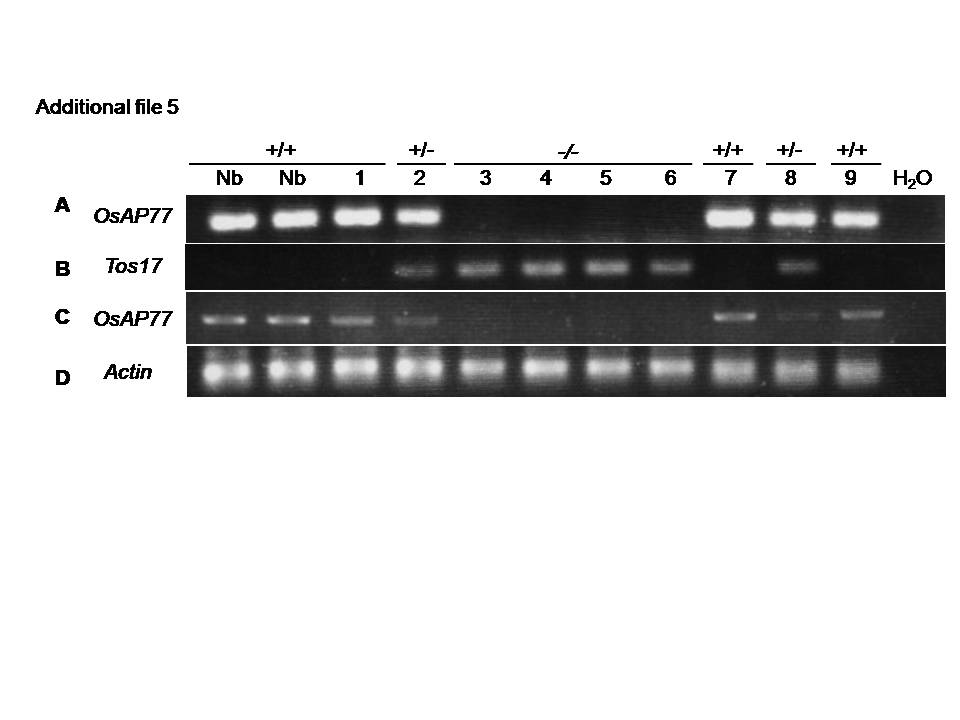

Supplement: Supplementary file 5 — Additional file 5:Quantitative measurement of GUS activity in leaves. The detached leaves of T3 plants were wounded by a needle-inoculation with/without droplets of conidia suspension (2×10^5^ conidia ml−1) of avrMO (A)/virMO (B) or VirXoo (B) at OD600 of 0.3. Then at the indicated times leaves were collected frozen by liquid nitrogen. The bars represent measurements averaged across the 3 samples from each transgenic line and repeated three times. Samples with asterisk in each parameter are significantly different: p < 0.05. (JPEG 39 KB) [file 12284_2014_9_MOESM5_ESM.jpeg]

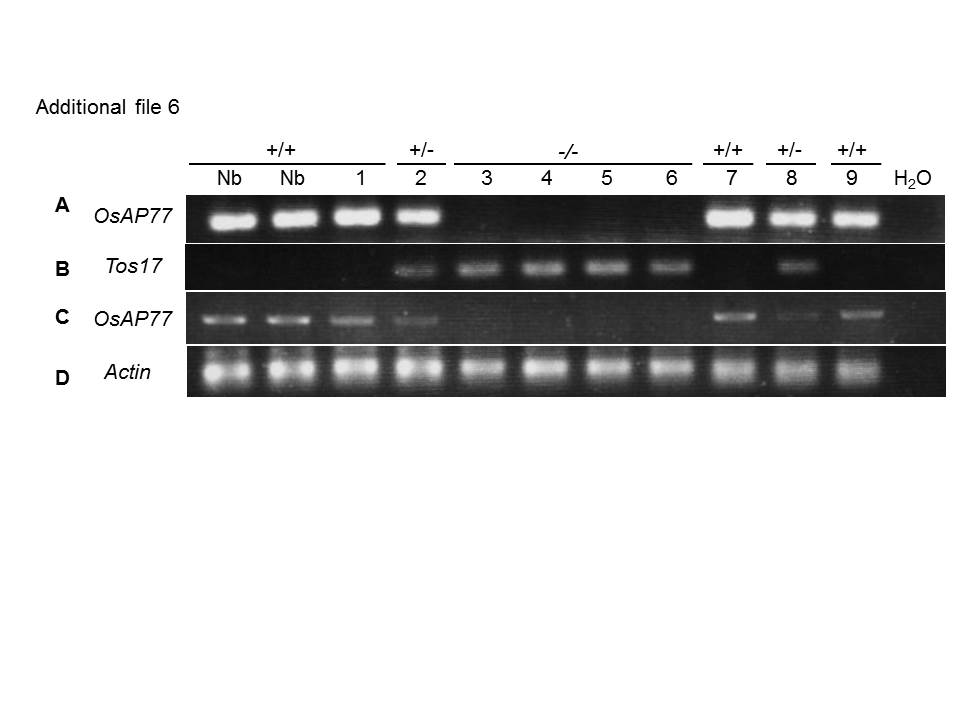

Supplement: Supplementary file 6 — Additional file 6:Analysis of progeny of OsAP77 mutant line. (A), (B) Genotyping using two sets of primers AP77P-5′/AP77P-3′ and Tos17-5′/AP77P-3′, respectively. Nb, Nipponbare; 1–9, progeny of the mutant line; H2O, negative control. +/+, OsAP77 (+/+); +/−, OsAP77 (+/−); −/− OsAP77 (−/−). (C) The expression level of the OsAP77 in the mutant M1 progeny and wild type plants by RT-PCR with primers, OsAP-5′/OsAP-3′. Total RNA was extracted from leaves from individual plants and used for RT-PCR. (D) The actin gene was used as the standard control to show the normalization of the amount of templates in PCR reactions. (JPEG 33 KB) [file 12284_2014_9_MOESM6_ESM.jpeg]

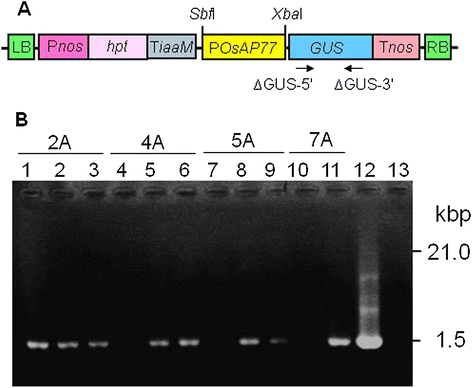

Supplement: Supplementary file 7 — Authors’ original file for figure 1 [file 12284_2014_9_MOESM7_ESM.gif]

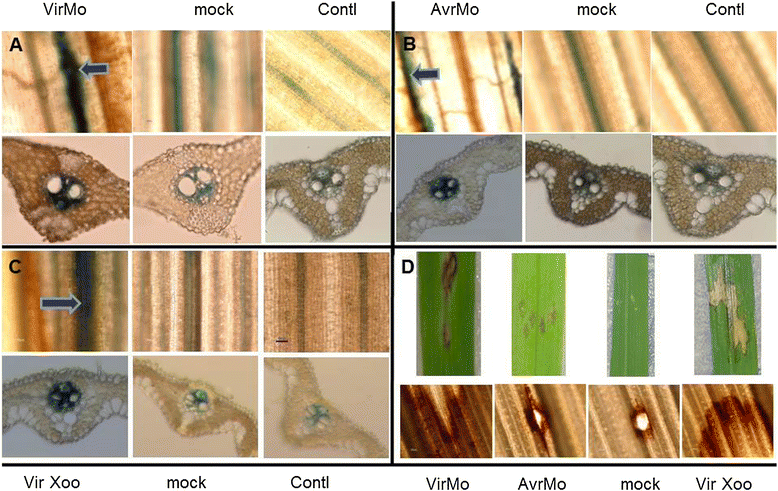

Supplement: Supplementary file 8 — Authors’ original file for figure 2 [file 12284_2014_9_MOESM8_ESM.gif]

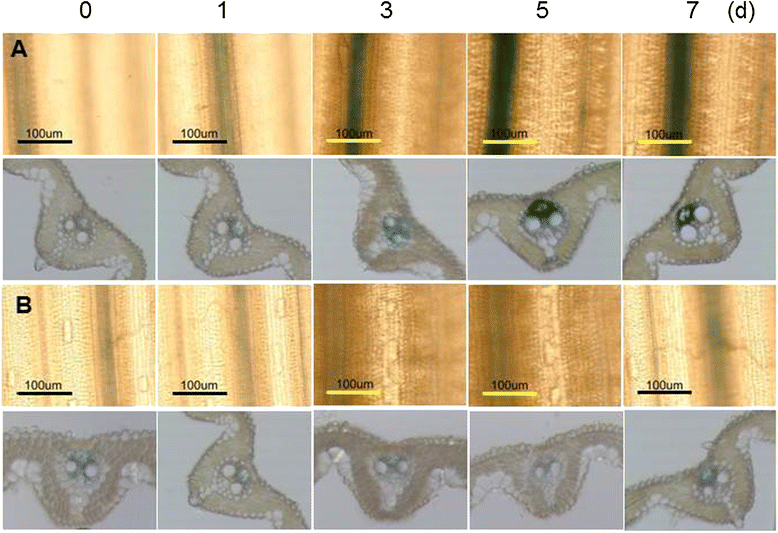

Supplement: Supplementary file 9 — Authors’ original file for figure 3 [file 12284_2014_9_MOESM9_ESM.gif]

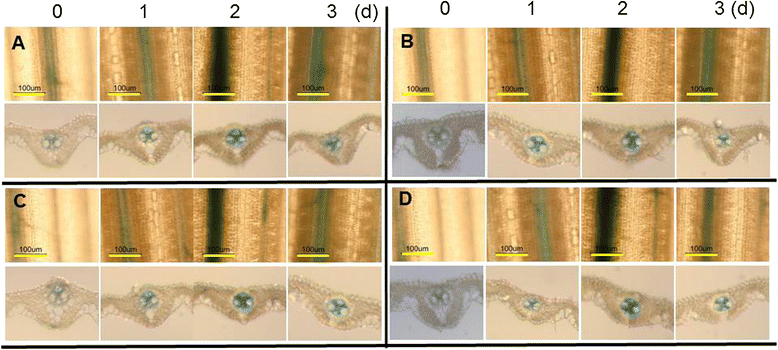

Supplement: Supplementary file 10 — Authors’ original file for figure 4 [file 12284_2014_9_MOESM10_ESM.gif]

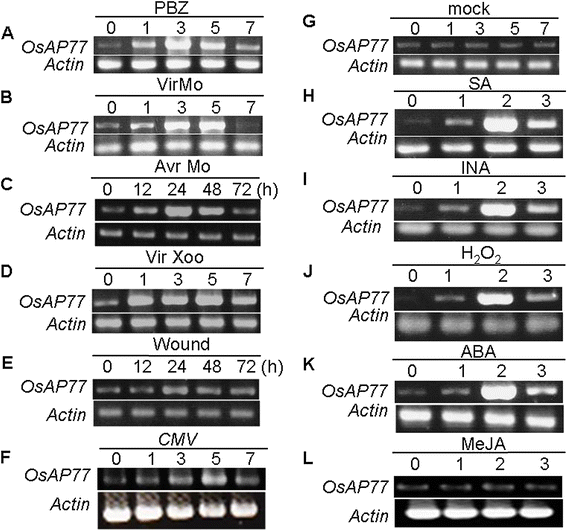

Supplement: Supplementary file 11 — Authors’ original file for figure 5 [file 12284_2014_9_MOESM11_ESM.gif]

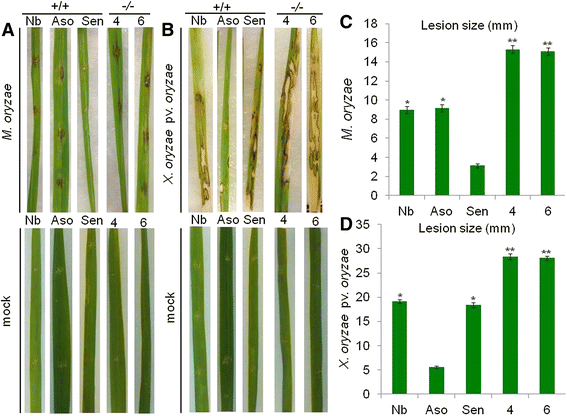

Supplement: Supplementary file 12 — Authors’ original file for figure 6 [file 12284_2014_9_MOESM12_ESM.gif]

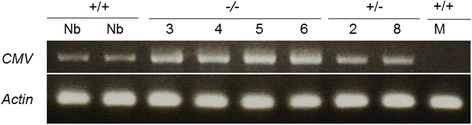

Supplement: Supplementary file 13 — Authors’ original file for figure 7 [file 12284_2014_9_MOESM13_ESM.gif]
